# MiR-145 Expression Accelerates Esophageal Adenocarcinoma Progression by Enhancing Cell Invasion and Anoikis Resistance

**DOI:** 10.1371/journal.pone.0115589

**Published:** 2014-12-31

**Authors:** Mathieu Francois Derouet, Geoffrey Liu, Gail Elizabeth Darling

**Affiliations:** 1 Latner Thoracic Surgery Research Laboratories, Toronto Discovery Medical Tower, University Health Network, Toronto, Ontario, Canada; 2 Department of Medical Oncology, Princess Margaret Hospital, University Health Network Toronto, Ontario, Canada; 3 Division of Thoracic Surgery, Toronto General Hospital, University Health Network, Toronto, Ontario, Canada; Chinese Academy of Medical Sciences, China

## Abstract

**Background:**

Carcinoma of the esophagus has a high case fatality ratio and is now the 6th most common cause of cancer deaths in the world. We previously conducted a study to profile the expression of miRNAs in esophageal adenocarcinoma (EAC) pre and post induction therapy. Of the miRNAs differentially expressed post induction chemoradiation, miR-145, a known tumor suppressor miRNA, was upregulated 8-fold following induction therapy, however, its expression was associated with shorter disease-free survival. This unexpected result was explored in this current study.

**Methods:**

In order to study the role of miR-145 in EAC, miRNA-145 was overexpressed in 3 EAC cell lines (OE33, FLO-1, SK-GT-4) and one ESCC cell line (KYSE-410). After validation of the expression of miR-145, hallmarks of cancer such as cell proliferation, resistance to chemotherapy drugs or anoikis, and cell invasion were analyzed.

**Results:**

There were no differences in cell proliferation and 5 FU resistance between miR145 cell lines and the control cell lines. miR-145 expression also had no effect on cisplatin resistance in two of three cell lines (OE33 and FLO-1), but miR-145 appeared to protect SK-GT-4 cells against cisplatin treatment. However, there was a significant difference in cell invasion, cell adhesion and resistance to anoikis. All three EAC miR-145 cell lines invaded more than their respective controls. Similarly, OE33 and SK-GT-4 miR-145 cell lines were able to survive longer in a suspension state.

**Discussion:**

While expression of miR-145 in ESCC stopped proliferation and invasion, expression of miR-145 in EAC cells enhanced invasion and anoikis resistance. Although more work is required to understand how miR-145 conveys these effects, expression of miR-145 appears to promote EAC progression by enhancing invasion and protection against anoikis, which could in turn facilitate distant metastasis.

## Introduction

Carcinoma of the esophagus has a high case fatality ratio. Over the past 20 years, the incidence of the esophageal adenocarcinoma (EAC) subtype has been increasing in North America and Europe [1]. Esophageal cancer has now become the eighth most common cancer and the sixth most common cause of cancer death in the world [2]. This dramatic increase has been associated with gastroesophageal reflux disease (GERD), obesity and Barrett's esophagus, which increases the risk of esophageal adenocarcinoma by 30-fold [3].

Recent literature has highlighted the role of microRNA (miRNA) in cancer progression and chemotherapy resistance. miRNAs have been shown to play an important role in the regulation of cell differentiation, proliferation and apoptosis [4]–[7]. As deregulation of these processes are features of cancer, it is likely that miRNAs play a role in carcinogenesis. Previous studies have reported that the expression of miRNAs is altered in cancers, linking miRNA expression to either initiation or progression of various cancers such as breast, lung, pancreas, prostate and CLL [8].

As miRNAs may act as tumor oncogenes or tumor suppressors [6], they are potential cancer biomarkers. We previously reported on the miRNA profile of esophageal cancer before and after neoadjuvant therapy [9]. We found 568 miRNAs, which were significantly up or down-regulated after neoadjuvant therapy. We also established that post-treatment high levels of miR-135b and miR-145 (which were induced by neoadjuvant therapy) were linked to a shorter disease-free survival. miR-135b has been described in the literature as a tumor promoter. It plays a central role in colorectal cancer progression and promotes metastasis in lung cancer [10]–[11]. However, miR-145 has been described as a tumor suppressor miRNA in a variety of cancers such as breast, lung, colon and stomach [12]. In esophageal squamous cell carcinoma (ESCC), miR-145 expression is down-regulated whereas when it is expressed, it inhibits cell proliferation and cell invasion [13]–[14]. The role of miR-145 in EAC has not been previously reported but, it was surprising that miR-145 (a previously identified tumor suppressor miR) expression was linked to worse prognosis in our EAC patients.

Herein, we investigate the role of miR-145 in EAC and the link between its expression and shorter disease free survival in patients. We hypothesized that overexpression of miR-145 in EAC may lead to reduced survival by increasing the metastatic potential of EAC cells.

## Materials and Methods

### Cell lines

OE33 (EAC), FLO-1 (EAC), SK-GT-4 (EAC) and KYSE-410 (ESCC) cell lines were purchased from the European Collection of Cell Cultures (ECACC, UK). All cells lines were cultured in RPMI 1640 with 10% FCS and 1 mM L-Glutamine and Penicillin/Streptomycin at 37°C and 5% CO_2_.

### miR plasmid transfection

Cells were plated (10^5^/well) in a 6 well plate 24 h prior to transfection. 1 µg of pcmv-miR and pcmv-miR-145 plasmid (Origene, USA) in 200 µl of Jet–Prime buffer (Polyplus, France) were mixed with 2 µl of Jet Prime transfection reagent (Polyplus, France) and allowed to incubate 10 mins at room temperature. 200 µl of this mixture was then added to the cells in 1.8 mL of RPMI 1640 with 10%FCS and incubated 48 h. After 48 h, the medium was changed to RPMI with 10%FCS and 0.5 mg/mL G418. The medium was changed every 3 days and the transfected cells were selected for at 14 days.

### Cell proliferation and Cisplatin/5-FU treatment

Cells were plated at 5×10^4^/well in a 6 well plate. After 48 and 96 h of incubation, cells were trypsonized and resuspended in 1 ml of RPMI 1640 with 10%FCS. Cells were then counted with a haemocytometer using the trypan blue exclusion method.

For cisplatin and 5-fluorouracil (5-FU) treatment (both drugs commonly used to treat esophageal cancer), cells were incubated with 5 µM cisplatin (Sigma) (resuspended in 0.9% NaCl solution) or 35 µM 5-FU (Sigma) (resuspended in DMSO) for 72 h. Cells were then washed, trypsonized and counted as described previously.

### RT-PCR

Total RNA was extracted using the miRVANA kit (Ambion, TX, USA) following the manufacturer's instructions. RNA samples from patients were previously isolated [9]. Firstly, cDNA was synthesized using miR Taqman RT probes (Applied Biosystems). The Real Time PCR was performed using miR PCR Taqman primers and Taqman Master Mix, No UNG following manufacturer's instructions (Applied Biosystems). Samples were run on an ABI 7800 HT machine. Expression of miR-145 was calculated as a relative expression ratio of the control (RNU6B) using the ΔΔCt method. The miR-145 and RNU6B Taqman primers were purchased from Applied Biosystems.

### Western Blotting

Cells were washed and then lysed using RIPA buffer. Proteins were then separated on an SDS-polyacrylamide gel (Bio-Rad) and then transferred to a PVDF membrane using a Semi Dry Blotting system (Bio-Rad). Membranes were then incubated overnight at 4°C with primary antibodies. The membranes were then washed and incubated with secondary HRP antibody for 1 h at room temperature. The membranes were then incubated with Super Signal West Pico chemiluminescent substrate kit (Thermo Scientific) and processed using a Bio-Rad developer. PARP ((46D11) Rabbit mAb #9532, dilution 1∶5000), Beta-Actin Rabbit mAb #4967, dilution 1∶5000), Cleaved Caspase 3 (Asp175) (5A1E) Rabbit mAb #96, dilution 1∶1000 and anti-rabbit HRP ((#7074) dilution 1∶5000) antibodies were purchased from Cell Signaling.

### Cell Adhesion Assay

10^6^ cells were plated in a well (6 well plate) pre-coated with either 15 µg/mL Fibronectin or 2 µg/mLVitronectin (R&D systems). Cells were incubated for 30 mins at 37°C with 5% CO_2_. Detached cells were then counted as described previously.

### Cell Invasion Assay

Cell invasion was assessed using CytoSelect Cell invasion Assay, Basement Membrane (Cell Biolabs, USA) following manufacturer's protocol. 5×10^4^ cells in RPMI were incubated 24 h on top of RPMI 1640 with 10%FCS. The invading cell number was determined using a standard curve.

### Wound Healing Assay

Cells were cultured in 6-well plates and grown in RPMI 1640 with 10%FCS until cell monolayer approached confluence then a scratch was made using a plastic pipette tip to produce a wound in each well. The cultures were photographed before and then 8 h and 24 h after incubation at 37°C. The length of the wound was measured.

### Anoikis Assay

10^5^ cells were plated in Low Adhesion plates (Corning) and cultured for 72 h at 37°C. After 72 h, cells were harvested and lysed with RIPA buffer. Level of anoikis was assessed by Western Blotting looking at the cleavage of PARP and caspase 3.

### Clonogenic Survival Assay

1000 cells were plated in either monolayer or Low Adhesion plates (Corning). After 72 h culture, the suspension cultures were centrifuged, trypsonized and replated. Cells were allowed to grow for 10 days. Colonies were then stained using Crystal violet staining and counted. The percentage survival was established by ratio the number of colonies in control and the number of colonies after suspension culture.

### Statistical analysis

All data were presented as the means ± SE from at least two independent experiments. Each sample was done in triplicate. Statistical analysis was performed by Student's t test or Mann Whitney test. Statistic significance is shown as *, meaning p<0.05.

## Results

### Verification of miR-145 expression in the esophageal cell lines

We previously described that neoadjuvant therapy induced miR-145 expression in esophageal cancer patients [9]. This result was obtained by microarray, therefore, we needed to confirm this results by RT-PCR (S1A Fig.). The levels of miR-145 in post treatment biopsies were around 100 fold higher than the pre-treatment biopsies.

So, in order to mimic the results obtained in patients samples subjected to neoadjuvant therapy, pre-miR-145 and control (pcmv) plasmid were transfected into OE33, FLO-1, SK-GT-4 and KYSE-410. After antibiotic selection, total miRNA collected and run through Real Time PCR to validate the expression of miR-145. In all cell lines, miR-145 expression was at least 1000 times higher than the corresponding cell lines (S1B Fig.).

### miR-145 inhibits cell proliferation, wound healing and enhance anoikis of esophageal squamous cell carcinoma

**Figure 1:**
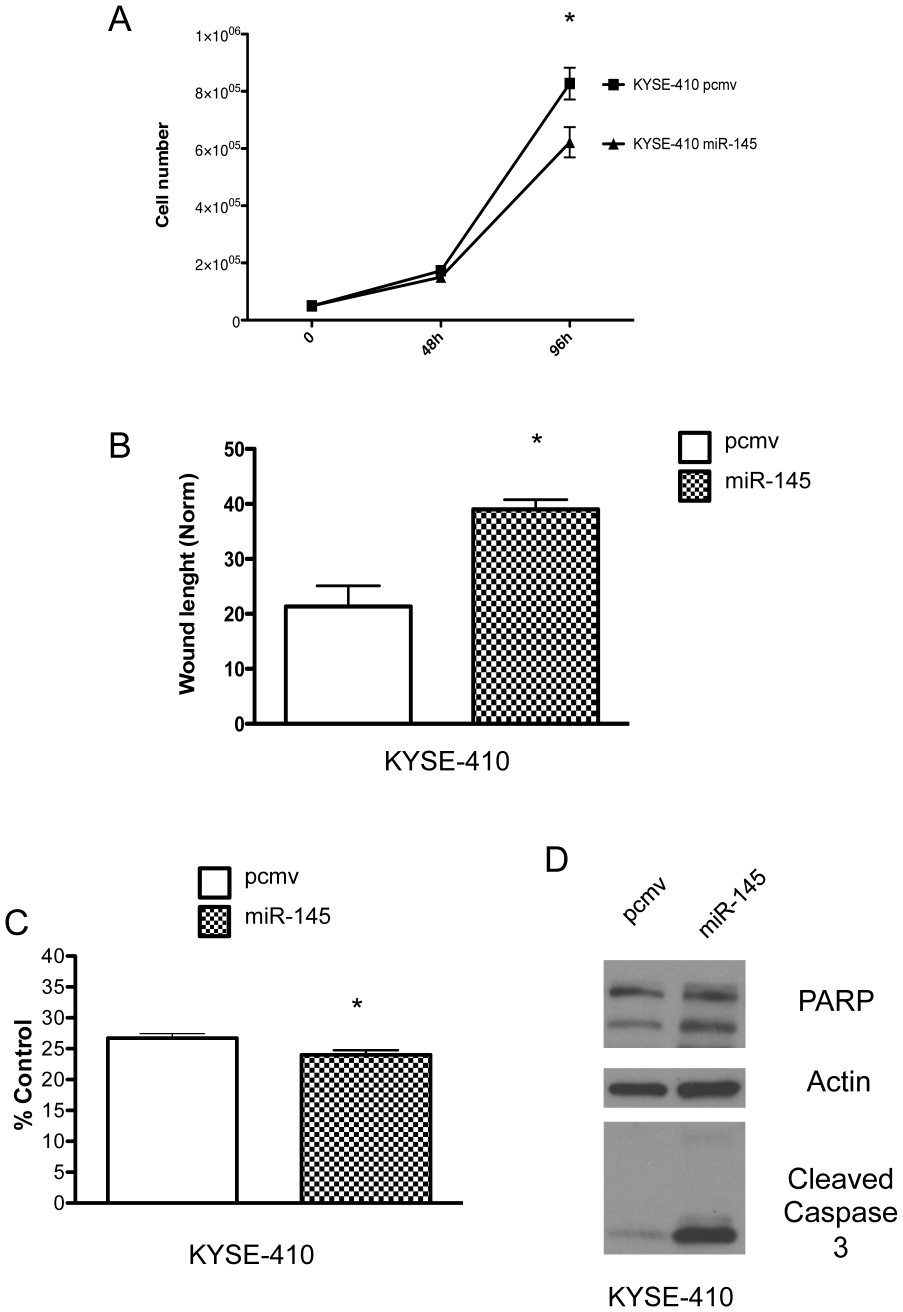
MiR-145 inhibited cell proliferation, delayed wound closure and enhanced anoikis in ESCC cells. (A) Cell proliferation and (B) wound healing assay of KYSE-410 pcmv and KYSE-410 miR-145 cells. miR-145 expression in KYSE-410 led to decreased numbers of colonies after cell suspension culture (C) and enhanced PARP and caspase 3 cleavage (D).*: p<0.05, n = 3.

**Figure 2:**
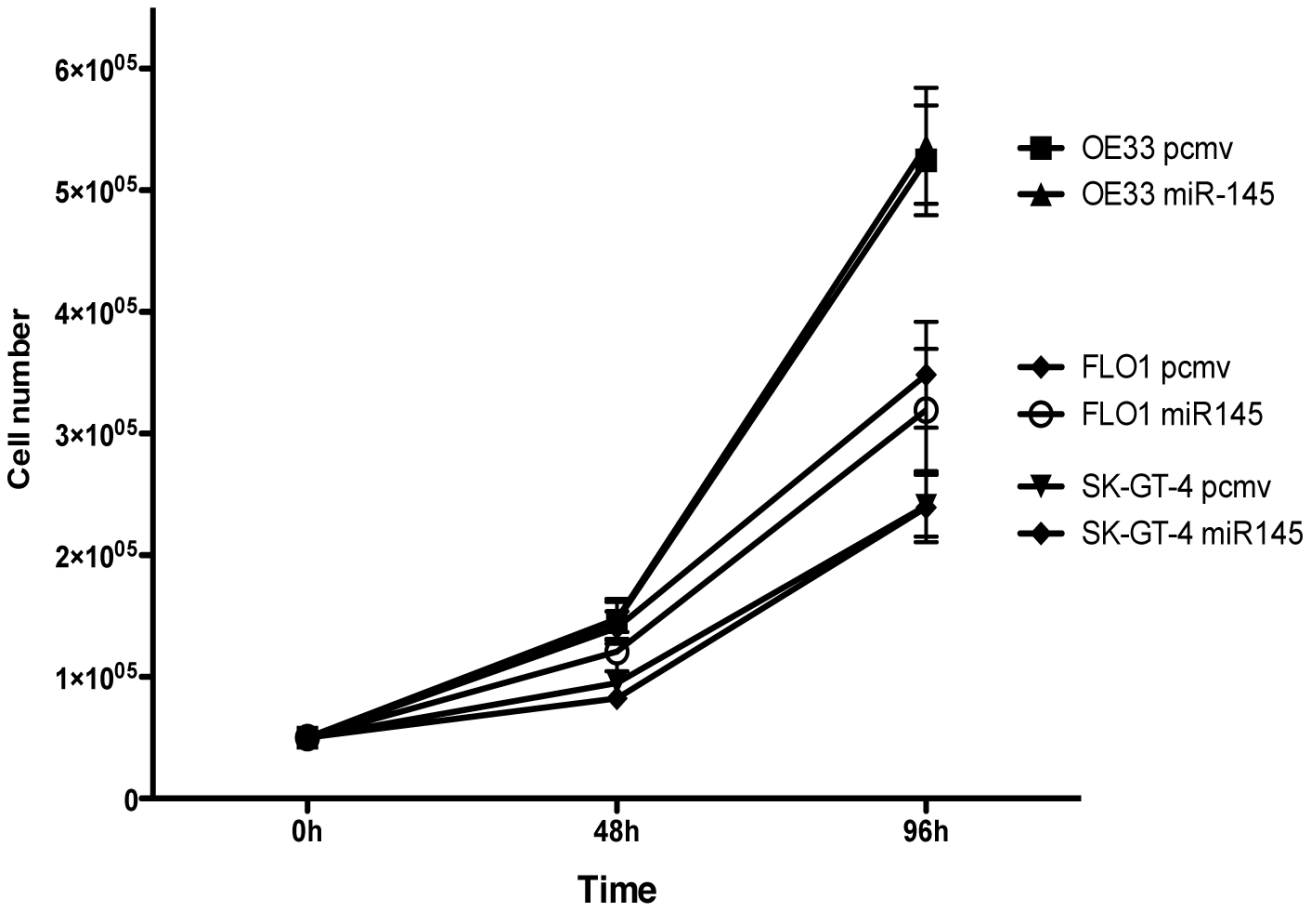
MiR-145 expression did not affect EAC cells proliferation. Cell proliferation assay of the pcmv and miR-145 EAC cell lines, n = 3

Since our patient population included both ESCC and EAC histologic subtypes, we first decided to investigate the effects of miR-145 in ESCC. We expressed miR-145 in an ESCC cell line, KYSE-410 (S1B Fig.). The expression of miR-145 in KYSE-410 cell line inhibited cell proliferation (Fig. 1A) as previously described. It also slowed down wound closure (Fig. 2B, S2A Fig.). Since miR-145 has been described to prevent metastasis [14], we decided to investigate if miR-145 can affect cell survival upon detachment. The ability to survive in a detached state is a requirement for metastasis. As a cell detaches from the extracellular matrix, it triggers cell death. Cell death triggered by detachment is known as anoikis and we can measure its rate by assessing the cleavage of PARP and caspases. miR-145 expression in KYSE-410 cell line resulted in more cleavage of PARP and caspase 3 during suspension culture (Fig. 2D). This effect on anoikis, along with its effect on proliferation, could potentially explain the decrease in number of colonies formed after suspension culture (Fig. 2C, S2B Fig.). These results confirmed previous reports that highlighted the tumor suppressor ability of miR-145 in ESCC [12]–[14].

### miR-145 does not affect cell proliferation of EAC cell lines

mir-145 expression had no effect on any of the 3 EAC cell lines in the cell proliferation assay. The proliferative rate of miR-145 expressing cell lines was similar to the pcmv cell lines (Fig. 2).

### miR-145 expression enhances cell adhesion to fibronectin but not to vitronectin

**Figure 3:**
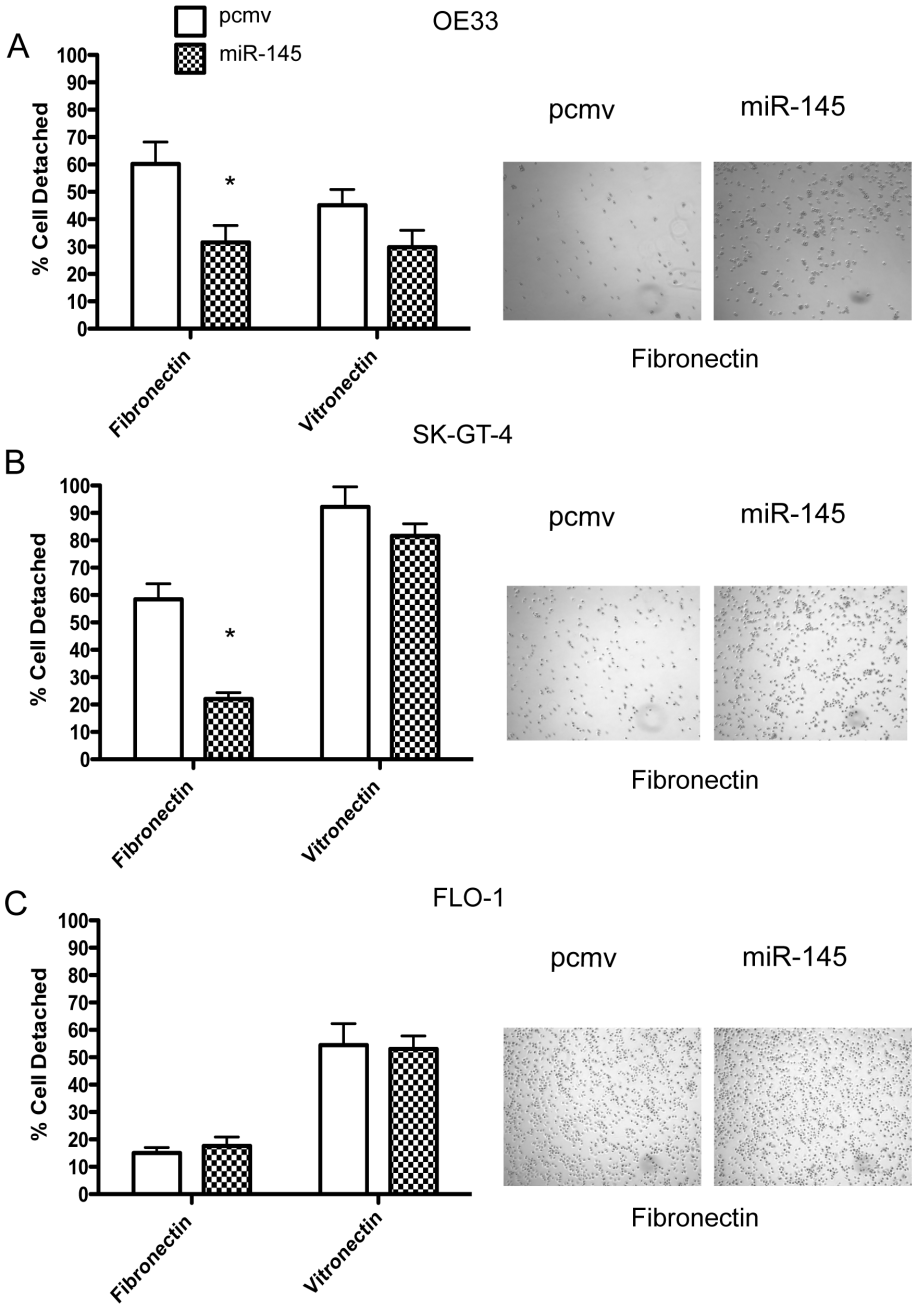
MiR-145 enhanced cell adhesion to fibronectin but not vitronectin. OE33 (A), SK-GT-4 (B) and FLO-1 (C) were plated onto plates pre-treated with either fibronectin or vitronectin. After 30 mins, the detached cells were counted. *: p<0.05, n = 3.

Evaluation of cell adhesion to vitronectin-coated plates showed no significant difference between the miR-145 and controls for all 3 EAC cell lines (Fig. 3A, B, C and S3 Fig.). However, in the fibronectin-coated plates, both OE33 miR-145 and SK-GT-4 miR-145 attached faster than their respective controls (Fig. 3A, C, S3 Fig.). No significant difference was observed in the FLO-1 cell line (Fig. 3B, S3 Fig.).

### miR-145 expression enhances wound healing and cell invasion

**Figure 4:**
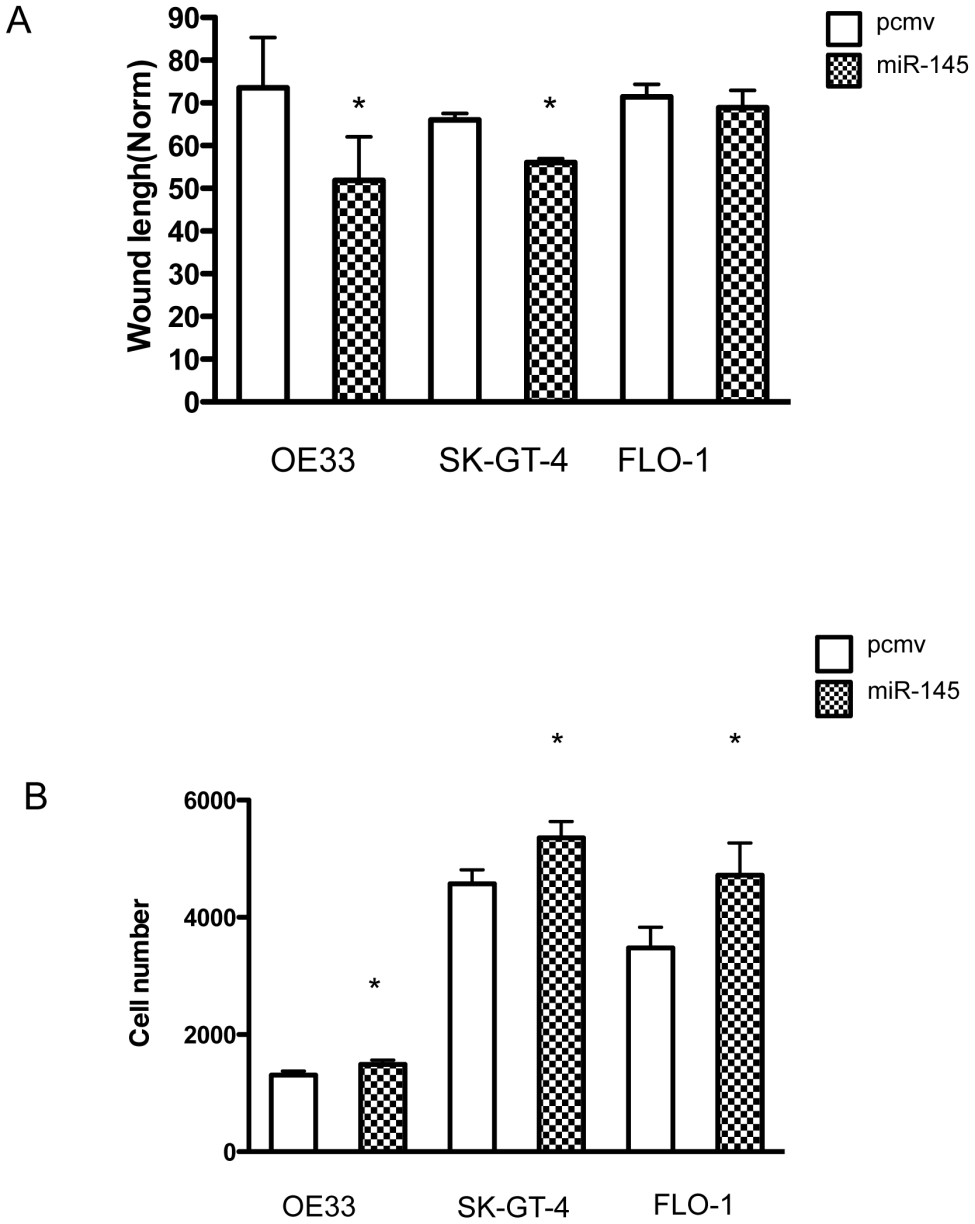
MiR-145 accelerated wound closure and enhanced cell invasion. Wound healing assay (A) with pcmv and miR-145 cell lines. The wound length was assessed after 8 h culture. *: p<0.05, n = 3. Cell invasion assay with pcmv and miR-145 cell lines (B). *: p<0.05, n = 3.

After 8 h of incubation, both OE33 and SK-GT-4 miR-145 cells closed the wound faster than their respective controls (Fig. 4A, S3 Fig.). No significant difference was observed with FLO-1 (Fig. 4A, S3 Fig.). After 24 h incubation, the wounds in FLO-1 and SK-GT-4 were closed for both pcmv and miR-145 and no significant difference was observed in OE33.

The miR-145-expressing EAC cell lines showed a significantly higher ability to invade compared to the control cell line (Fig. 4B). The effect on cell invasion was more moderate for OE33 when compared to FLO-1 and SK-GT-4 but was still significant.

### miR-145 expression protects EAC cells against anoikis but not against chemotherapy drugs

Anoikis resistance is an important step in cancer progression. The ability to survive in a detached state allows cells to migrate through the blood stream and create distant metastases. Since we already tested miR-145 effect on anoikis in an ESCC model, we tested if expression of miR-145 could affect EAC anoikis resistance.

**Figure 5:**
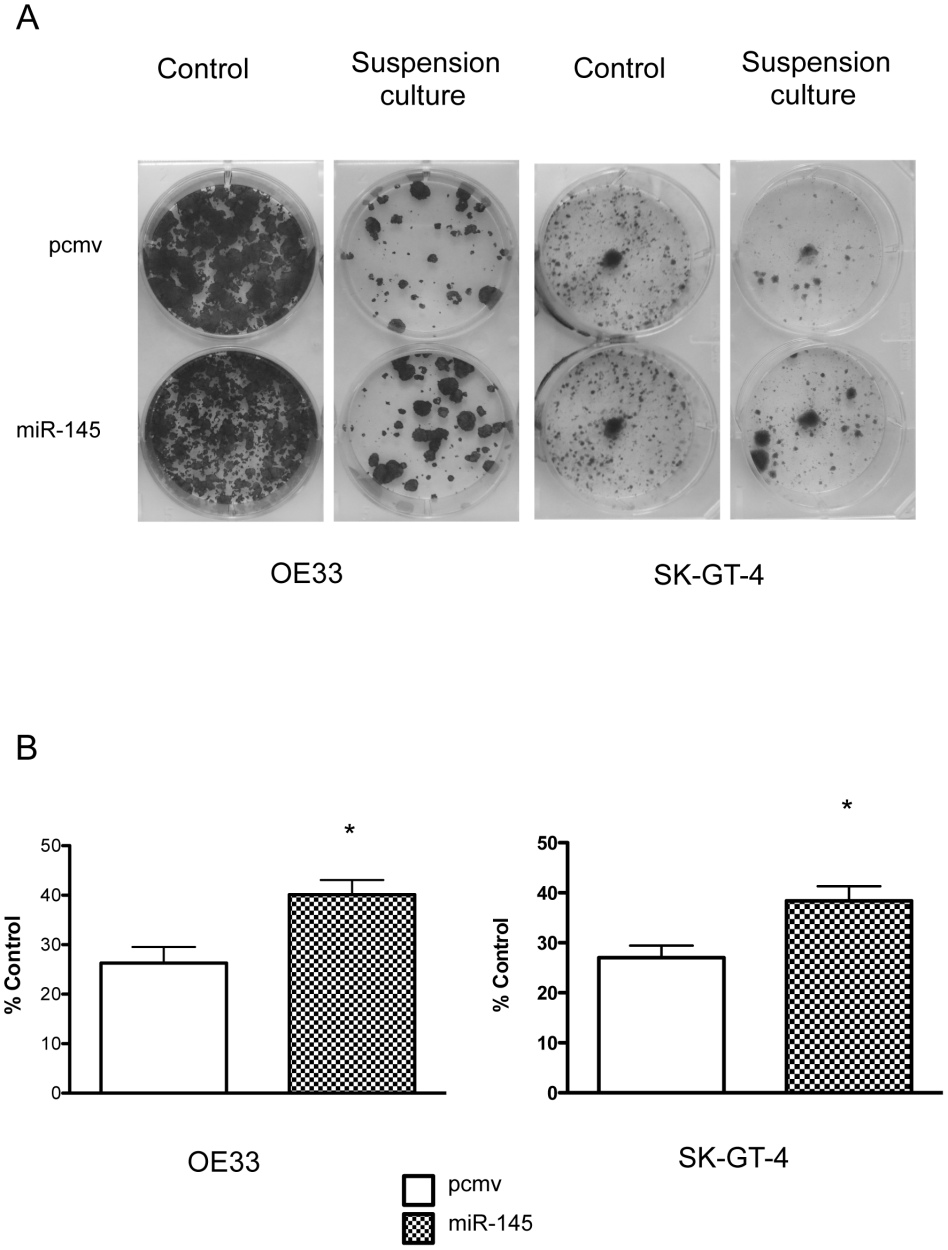
MiR-145 enhanced the clonogenic potential of OE33 and SK-GT-4 after suspension culture. Photo images of clonogenic assay after 72 h suspension culture with OE33 and SK-GT-4 (A). Results showed the average percentage of colonies formed after suspension culture compare to the number of colonies formed in monolayer (B). *: p<0.05, n = 2.

We first measured the effect of miR-145 on a colony forming assay after suspension culture. miR-145 cells were able to create more colonies after 72 h suspension culture than the pcmv cells (Fig. 5A, B). FLO-1 cells were not able to form colonies.

**Figure 6:**
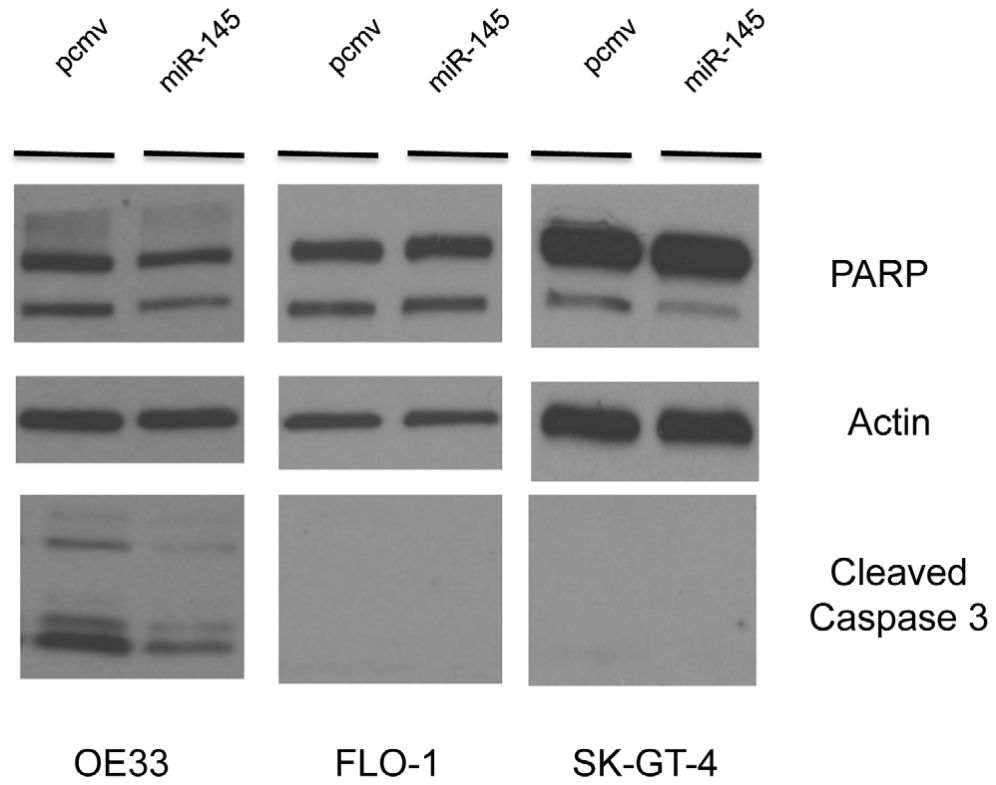
MiR-145 protected EAC cells against anoikis. pcmv and miR-145 EAC cell lines were cultured in suspension for 72 h. The levels of cleaved PARP and cleaved caspase 3 were assessed by Western Blotting.

Since miR-145 has no effect on EAC cell proliferation, we speculate that miR-145 protected the cells against anoikis, enhancing cell survival in a detached state. We therefore measured anoikis levels in the EAC cell lines. In OE33 and SK-GT-4 cell lines, the control cells (pcmv) revealed more PARP cleavage than their miR-145 counterpart after 72 h suspension culture (Fig. 6). Furthermore, in OE33, miR-145 cells expressed less cleaved caspase 3 than the pcmv cells. However, the cleaved caspase 3 could not be detected in SK-GT-4 cells. There was no difference between pcmv and miR-145 in the FLO-1 cell line (Fig. 6). These results demonstrated that miR-145 expression delayed the cleavage of PARP and caspase 3 and by doing so is able to protect EAC cells against anoikis. So in regard to anoikis resistance, miR-145 has an opposite effect in EAC compared to an ESCC model.

**Figure 7:**
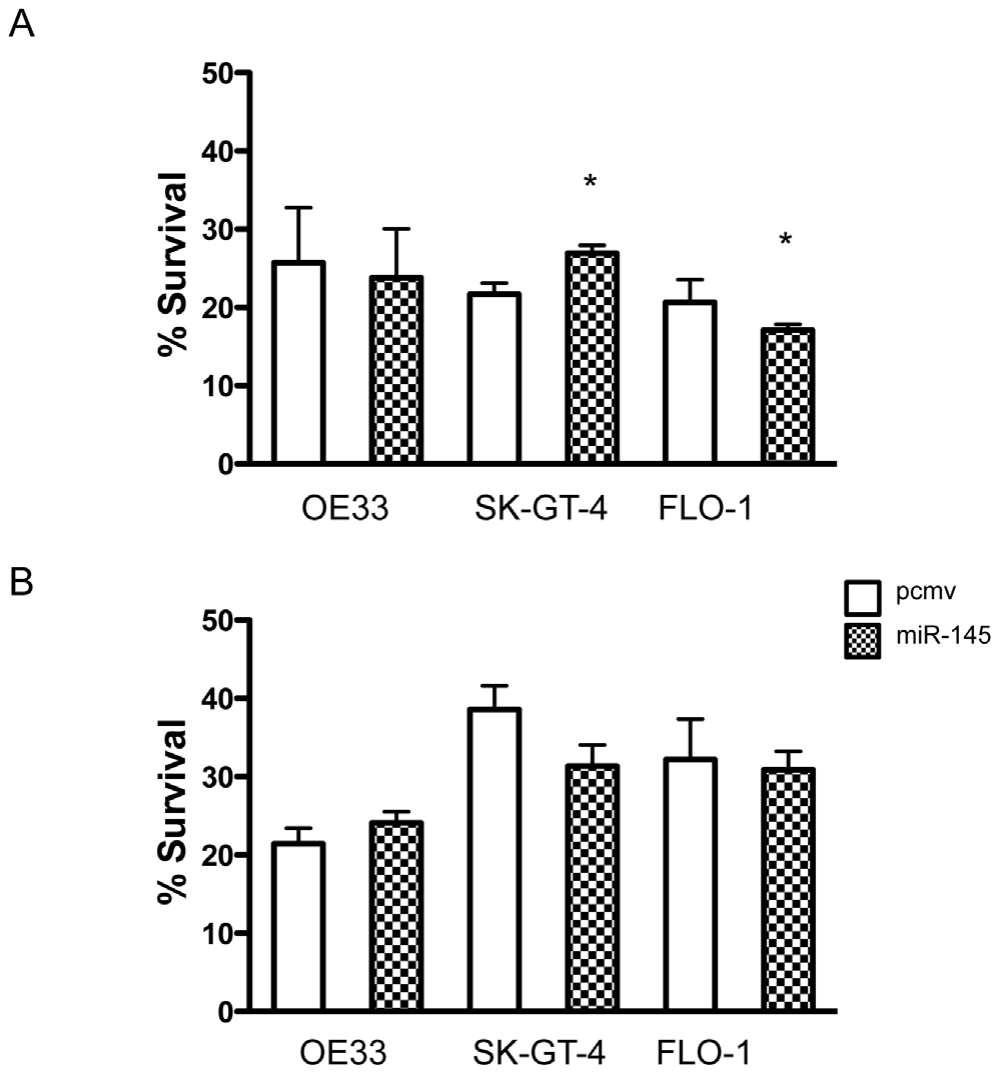
MiR-145 did not generally affect the EAC resistance to chemotherapy drugs. OE33, FLO-1 and SK-GT-4 (pcmv and miR-145) cells were cultured for 72 h with either (A) cisplatin (5 µM) or (B) 5-fluorouracil (35 µM) and their respective control. Live cell number was then assessed. Results show the percentage of live cells compared to the control treatment. *: p<0.05, n = 3.

Since anoikis is one form of apoptosis, we investigated if miR-145 could protect cells in a similar effect against other forms of apoptosis. When we tested whether miR-145 expression was associated with resistance to cisplatin or 5-FU resistance, we noticed that miR-145 did not protect OE33 against either cisplatin (Fig. 6A) or 5-FU (Fig. 7B). However, miR-145 protected SK-GT-4 cells against cisplatin but not against 5-FU (Fig. 7A, 7B). In FLO-1 cells, miR-145 enhanced the effect of cisplatin (Fig. 7A) but had no effect on 5-FU resistance (Fig. 7B). By protecting cells against anoikis, miR-145 allowed cells to remain alive longer in a detached state, which increased the local or distant metastasis efficiency.

## Discussion

In this study, we explored the effect of miR-145 expression in EAC and ESCC cell lines. Previous studies reported that miR-145 acts as a tumor suppressor in ESCC. Yet in our previous work, miR-145 overexpression was linked to worse disease-free survival in esophageal cancer patients. We hypothesized that miR-145 overexpression may increase the metastatic potential of EAC cells. Since the ability of cancer to metastasize is linked to its ability to proliferate, its survival in a detached state in the blood stream, its adherence to vascular endothelium and its ability to invade, we evaluated these properties in three EAC cell lines and one ESCC cell line. Cell lines were transfected with either miR-145 expressing plasmids or control plasmids. miR-145-expressing EAC cell lines exhibited increased cell adhesion, cell invasion and protection against anoikis. In contrast, when expressed in an ESCC cell line, miR-145 expression inhibited cell proliferation while sensitizing cells to anoikis. This latter finding is novel and supports previous reports that miR-145 act as a tumor suppressor in ESCC.

We found that expression of miR-145 in EAC cell lines does not affect cell proliferation or response to chemotherapy drugs such as cisplatin or 5-FU but miR-145 does enhance wound healing, cell adhesion to fibronectin, cell invasion and resistance to anoikis. This is the first report, which has studied the effects of miR-145 in EAC. Furthermore, the finding that the effect of miR-145 expression in EAC cell lines is opposite to its effect in ESCC cell lines is also novel.

This is not the first or only miRNA that has been described to be both pro and anti-tumorigenenic depending on the organ of expression. miR-125b has been described as an oncomiR in cancers such as prostate, thyroid [15]–[17] but its expression in ovarian and breast cancer is down-regulated, enhancing cell proliferation [18]–[20]. Similar observations have been made with several other miRNAs [21]. Our results reinforce the concept that the organ affected by cancer and the nature of the cancer could dictate the role of the miRNA. For example, OE33 and SK-GT-4, but not FLO-1, are EAC cell lines established from patients who had Barrett's esophagus. Others postulate that the staging of the cancer could also play a key role in influencing the miRNA abilities [22]. A recent report postulates that up regulation of miR-145 is associated with metastasis in colorectal cancer [23]. This observation goes against previous literature [24]–[25] but it could be potentially explained by the presence or absence of miRNA targets during cancer progression.

In our results, we showed that miR-145 expression leads to stronger cell adhesion to fibronectin, but not to vitronectin, which has been previously described in prostate cancer cells [26]. However, in the same paper, they showed that expression of miR-145 leads to decreased invasion whereas we described the opposite effect. The fact that cell adhesion is enhanced only to fibronectin could be an indication of the involvement of specific integrins. The α5β1 integrin is known to bind fibronectin [27]. In breast cancer, overexpression of α5β1 is associated with cancer cell survival [28] and tumor progression in lung cancer [29]. The interaction between fibronectin and α5β1 integrin has been shown to lead to activation of cell signaling pathways involved in cell invasion and metastasis [30]. Furthermore, α5β1 integrin expression has been associated with resistance to anoikis in intestinal epithelial cells and cervical cancer [31]–[32].

Therefore, miR-145 expression in EAC could increase α5β1integrin expression leading to adhesion to fibronectin, which will in turn, enhance cell invasion potential and anoikis resistance. If cell invasion ability is combined with anoikis resistance, most of the elements for a more efficient metastasis are present. Though we could speculate that EAC cells with high levels of miR-145 metastasize more efficiently, assessment in the clinical setting is still lacking.

In conclusion, expression of miR-145 in EAC cell lines generally results in increased cell adhesion to fibronectin, increased cell invasion and resistance to anoikis, but did not affect cell proliferation or response to chemotherapy drugs. When expressed in an ESCC cell line, miR-145 has the opposite effect of inhibiting cell proliferation and enhancing anoikis. More work is required to understand how miR-145 is induced during neoadjuvant therapy and which signaling pathways are required to generate its pro-metastatic effect, but measurement of miR-145 following neoadjuvant therapy could potentially be useful in identifying patients at risk of recurrence. In turn such patients could be selected for further chemotherapy.

## Supporting Information

S1 Fig **Validation of mir-145 expression in patients and cell lines.** (A) qRT-PCR results measuring miR-145 in patient biopsies. (B) qRT-PCR results measuring miR-145 in cell lines transfected with plasmid control (pcmv) or miR-145 (miR-145) for each cell line. *: p<0.05.
(TIF)

S2 Fig **MiR-145 expression in KYSE-410 slowed down wound healing and inhibited colony formation after suspension culture assay.** Photo images of a wound healing assay (A) and clonogenic assay (B) using KYSE-410 pcmv and miR-145 cells.
(TIF)

S3. Fig **MiR-145 expression in OE33 and SKGT-4, but not in FLO-1, helped the cells to close the wound faster.** Photo images of wound healing assay with OE33, SK-GT-4 and FLO-1. The photos were taken at 0 and 8 h.
(TIF)
