# Supplementary figures and images for: MiR-145 Expression Accelerates Esophageal Adenocarcinoma Progression by Enhancing Cell Invasion and Anoikis Resistance
Source: PLoS One. 2014 Dec 31;9(12):e115589. doi: 10.1371/journal.pone.0115589 (PMC4281214; doi:10.1371/journal.pone.0115589)

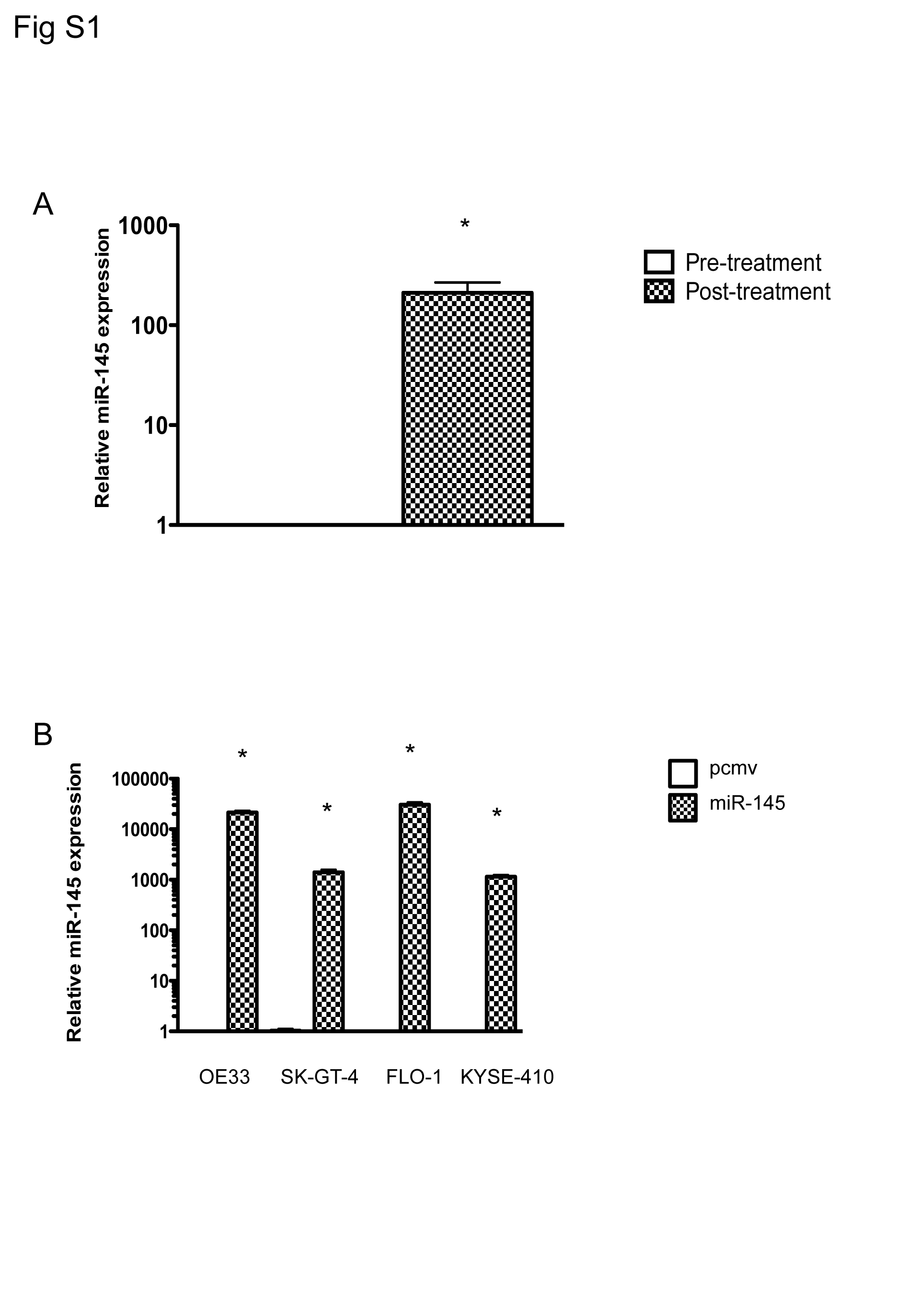

Supplement: S1 Fig — Validation of mir-145 expression in patients and cell lines. (A) qRT-PCR results measuring miR-145 in patient biopsies. (B) qRT-PCR results measuring miR-145 in cell lines transfected with plasmid control (pcmv) or miR-145 (miR-145) for each cell line. *: p<0.05. (TIF) [file pone.0115589.s001.tif]

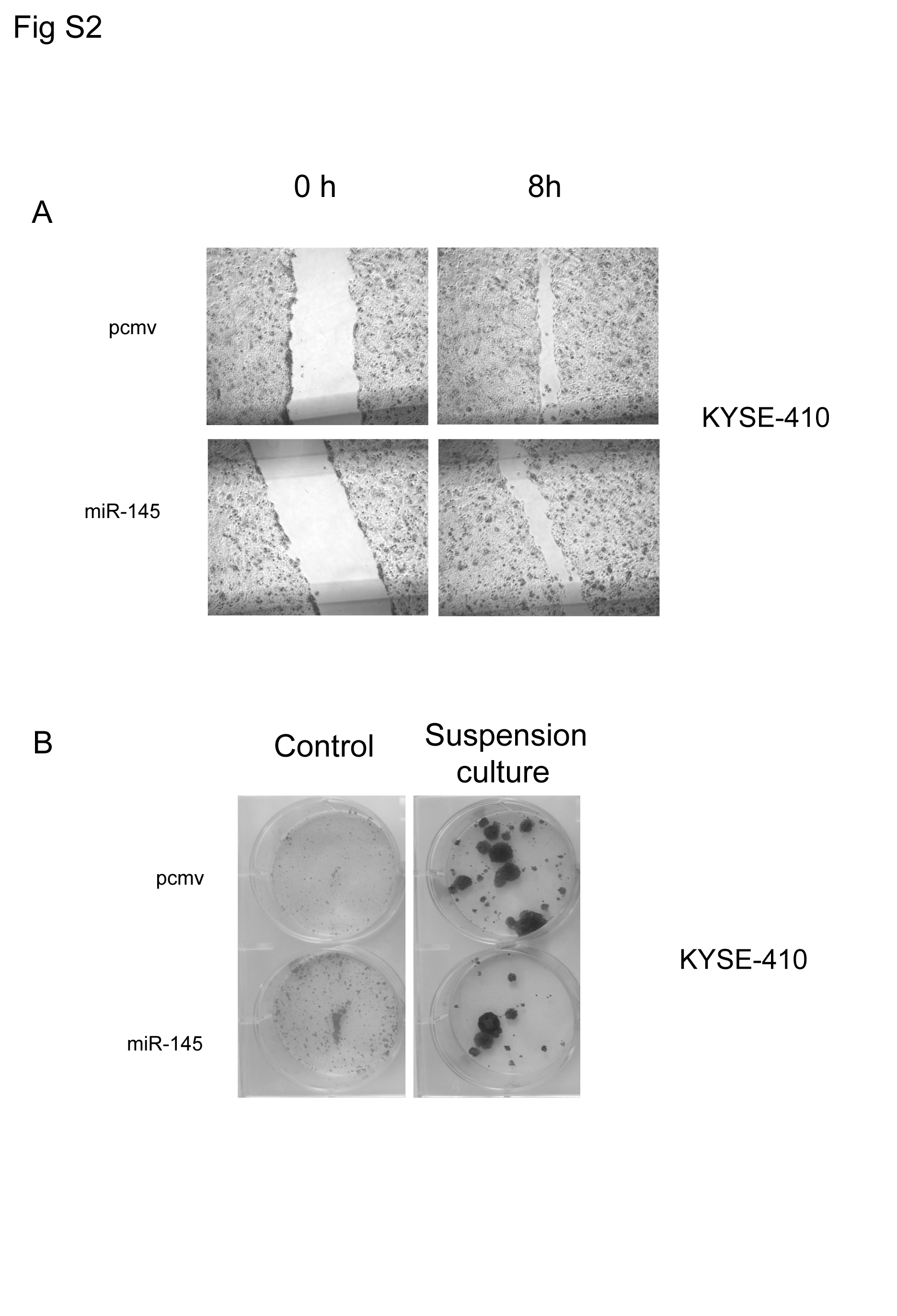

Supplement: S2 Fig — MiR-145 expression in KYSE-410 slowed down wound healing and inhibited colony formation after suspension culture assay. Photo images of a wound healing assay (A) and clonogenic assay (B) using KYSE-410 pcmv and miR-145 cells. (TIF) [file pone.0115589.s002.tif]

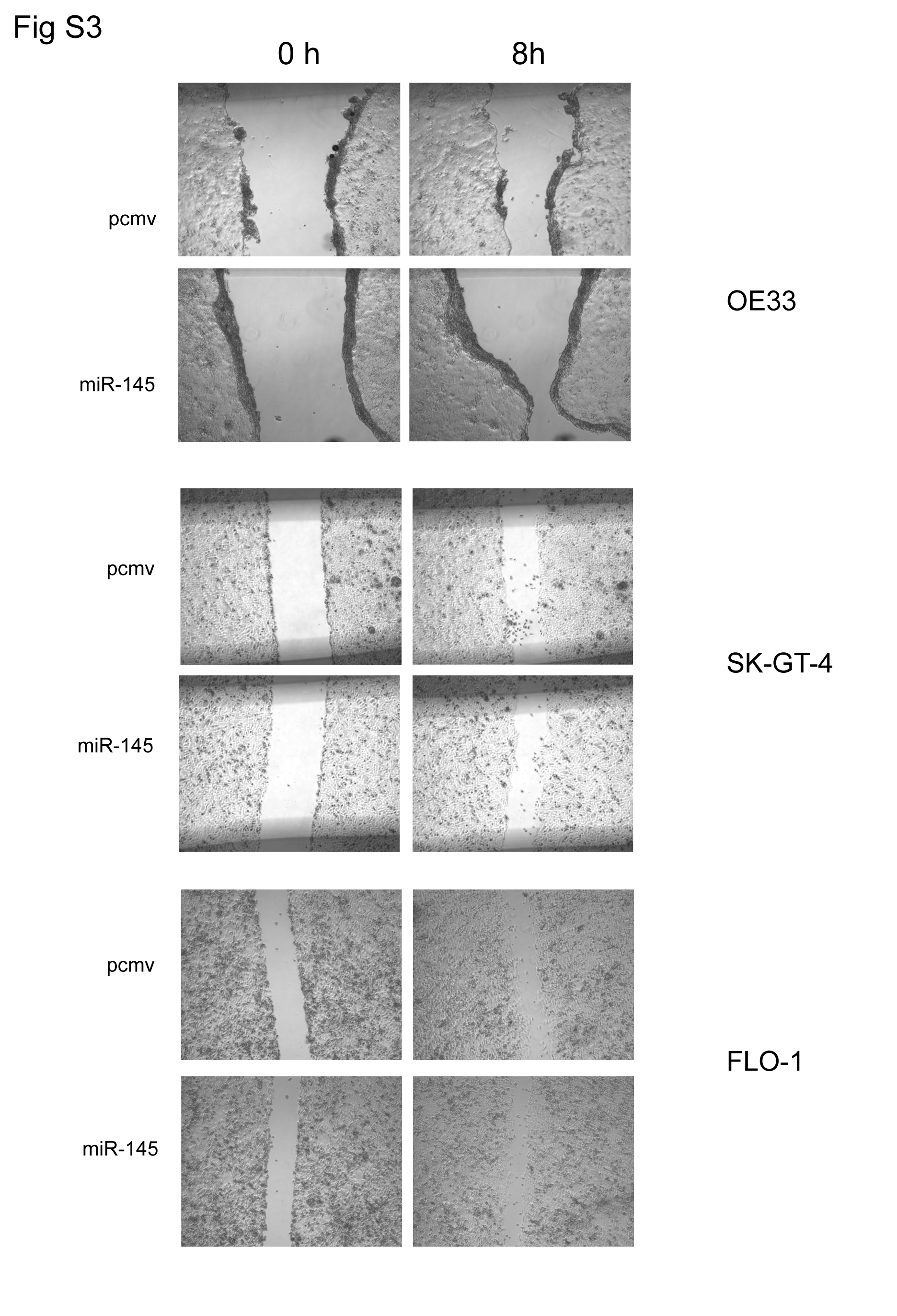

Supplement: S3. Fig — MiR-145 expression in OE33 and SKGT-4, but not in FLO-1, helped the cells to close the wound faster. Photo images of wound healing assay with OE33, SK-GT-4 and FLO-1. The photos were taken at 0 and 8 h. (TIF) [file pone.0115589.s003.tif]
